# Universal Grammar and Biological Variation: An EvoDevo Agenda for Comparative Biolinguistics

**DOI:** 10.1007/s13752-014-0164-0

**Published:** 2014-03-15

**Authors:** Antonio Benítez-Burraco, Cedric Boeckx

**Affiliations:** 1 Department of Spanish Philology and its Didactics, University of Huelva, Huelva, Spain; 2 Catalan Institute for Research and Advanced Studies (ICREA) & Department of Linguistics, University of Barcelona, Barcelona, Spain

**Keywords:** Biolinguistics, Evolutionary developmental biology (EvoDevo), Genetics, Language disorders, Variation

## Abstract

Recent advances in genetics and neurobiology have greatly increased the degree of variation that one finds in what is taken to provide the biological foundations of our species-specific linguistic capacities. In particular, this variation seems to cast doubt on the purportedly homogeneous nature of the language faculty traditionally captured by the concept of “Universal Grammar.” In this article we discuss what this new source of diversity reveals about the biological reality underlying Universal Grammar. Our discussion leads us to support (1) certain hypotheses advanced in evolutionary developmental biology that argue for the existence of robust biological mechanisms capable of canalizing variation at different levels, and (2) a bottom-up perspective on comparative cognition. We conclude by sketching future directions for what we call “comparative biolinguistics,” specifying which experimental directions may help us succeed in this new research avenue.

The present article is concerned with the amount and kind of variation that we think linguists and researchers in allied disciplines should wrestle with if they are to contribute to a proper characterization of the biological foundations of language and, in so doing, to a rapprochement of the cognitive sciences with the biological sciences, an enterprise that we refer to as *biolinguistics* (Di Sciullo and Boeckx 2011; Boeckx 2013; Boeckx and Grohmann 2013).

Our main contention in this article is that although the comparative method has figured prominently in linguistics, the objects routinely compared (languages, dialects, sociolects) may not be the only, or indeed the most appropriate ones to shed light on the biological foundations of our species-specific linguistic capacity. There are, we claim, deeper layers of variation to explore and to understand. Indeed, as we intend to show here, these deeper layers of variation beg questions regarding the proper biological interpretation of standard concepts in the field of (bio)linguistics, such as Universal Grammar (Chomsky 1965). We believe that in part it is the failure to properly address these sources of variation that has rendered the adjective “universal” (and in fact the whole argument of language as a specific component of human biology) more controversial than it should be.

At the same time, we also think, and will argue in what follows, that linguists have asked questions regarding the locus of linguistic variation (traditionally construed) that would be useful to extend to the layers of variation we are about to highlight. But for this to be successful, it is important to carefully select, and properly conceptualize, the tools one uses to establish comparisons. Here, we think it is imperative to formulate these tools at the right level of granularity to allow for interdisciplinary exchange, in line with Poeppel and Embick (2005). It is our conviction that once this is done, these tools and questions about variation would enrich the range of studies that currently constitutes what we may want to call “comparative biolinguistics,” to be distinguished from the traditional label of comparative linguistics, for reasons that will be spelled out in this article.

Our reflections are structured as follows. First, we will clarify why we think this new kind of linguistic variation is real, and problematic for standard characterizations of certain central concepts in the field of (bio)linguistics. In a nutshell, linguists routinely acknowledge, and, we believe, have quite successfully dealt with, linguistic variation at the surface (languages, dialects, sociolects, and the like). At the same time, they have usually regarded the faculty of language giving rise to these variants as uniform across the species (pathologies aside), and genetically determined. However, the recent emphasis on the biological underpinnings of language—the return of biolinguistic concerns—has begun to reveal deeper layers of variation down to the genetic level that make these standard assumptions unreasonable, and beg the question of where the uniformity or universality of grammar comes from.

We will then proceed to argue that in fact this deeper variation is problematic mainly for naive approaches to the biology of language. However, we find that these approaches are still the majority within the field, and should therefore be corrected to accommodate the variation we find.

We will then suggest that new theoretical approaches coming from biology, paradigmatically, evolutionary developmental biology (EvoDevo), could help us deal with, and account for, all the observed variation, while offering sources of uniformity to reliably give rise to species-typical linguistic capacities. As a matter of fact, we claim that certain EvoDevo concepts could contribute significantly to our understanding of the nature of language disorders.

Finally, we will sketch some concrete and novel ways in which linguists and other scientists in adjacent fields could contribute to a comparative biolinguistics. In this final section, we are led to point out some important limitations of recent tools used in this domain.

## Layers of Variation

Variation thoroughly pervades language. The human language faculty manifests itself in the form of many different languages, which are in turn (slightly) diverse across social groups, interactional contexts, geographical areas, and so on. Ultimately, differences can be found from one person to another, and even regarding the same person, for instance, when placed in different scenes. All of this is very familiar, and, we feel, linguists have fairly successfully coped with all this variation (which, of course, is not to say that everything is understood at this level). It is now clear that linguistic phenomena vary in systematic and constrained ways, and can be accounted for by the right mixture of general principles governing cognition and statistical biases (see Baker 2001; Labov 2001; Yang 2006; Pearl 2007; Biberauer 2008; Culbertson 2010, for accessible overviews and concrete proposals to capture this variation).

At the same time, the human faculty for language has routinely been assumed to be uniform within the species (Chomsky 1965, 1980), an assumption captured by the term “Universal Grammar.” For many people, this assumption is a central tenet of the Chomskyan revolution in the language sciences. As is well known, this revolution brought about a radical shift of focus in language studies, a shift oriented towards biology, and away from behaviorism; indeed, a shift that provides the foundations of modern biolinguistics.

Early investigations of the biological foundations of language (see Lenneberg 1967) relied on evidence from a variety of sources, such as:

1. The way in which language is acquired by the child, which suggests that language “learning” mechanisms are biased or constrained in certain ways.
2. The fact that specific language deficits recurrently appear whenever certain brain areas are impaired (either developmentally or after a stroke, a trauma, or a tumoral process).
3. Ultimately, the existence of language-related components in other extant or extinct species.

These arguments in favor of Universal Grammar continue to be made even by those who otherwise distance their positions from Chomsky’s in a number of ways (see, e.g., Jackendoff 2002, Chap. 4). Recent advances in neuroscience and molecular biology have allowed us to gain a better understanding of the biological underpinnings of language. For instance, we are now in a position to accurately know which brain areas and circuits are active during language processing (see Stemmer and Whitaker 2008; Friederici 2011; or Friederici and Gierhan 2013 for overviews). Similarly, we have identified many of the genes that contribute to the development and the initial wiring of these areas and circuits during growth (Benítez-Burraco 2009; Graham and Fisher 2012). Ultimately, the role of these brain areas and circuits, and of these genes in other extant (and even extinct) species are being studied, revealing (deep) homologues of (aspects of) the language faculty (see the contributions in Di Sciullo and Boeckx 2011; Boeckx and Grohmann 2013).

However, this revival of biolinguistic concerns has not yet substantially changed some of the concepts that remain at the center of the field, such as Universal Grammar. In the vast majorities of studies in biolinguistics (we will return to exceptions), what is called the “linguistic genotype,” i.e., the whole set of genes involved in ultimately allowing for language growth in the child, or “that part of our genetic endowment that is relevant to our linguistic development” (Anderson and Lightfoot 1999, p. 702), is assumed to be uniform across the species, pathologies aside. Moreover, we continue to come across many studies in which the “linguistic brain”—i.e., the whole set of brain areas and circuits involved in language processing—is expected to be equally organized and sharply defined in all individuals. For many, these “language areas” only process specific linguistic features and operations. Ultimately, the faculty of language is supposed to be equally implemented in all subjects as one of the modules encompassing their minds/brains. This module is further thought to be present ab initio, part of a genetic program of sorts (see, e.g., Wexler 1999 and Guasti 2002; and see Longa and Lorenzo 2008 for a critical overview of this “genocentric” literature).

Our reading of the growing literature on the biological foundations of language suggests to us that the state of affairs described in the previous paragraph is quite an erroneous, and in fact simplistic, view of reality. In particular, a fair amount of evidence exists that suggests that the human faculty for language is not really uniform. To be fair, some of this evidence is not new, but we feel that its significance has not yet been properly appreciated. Doing this is the primary purpose of our article.

When one thinks about the possibility of a variable implementation of the language faculty, one is likely to think about facts like: (1) different linguistic modalities can coexist in the same subject, as people bilingual in oral and sign languages nicely exemplify (Emmorey and McCullough 2009); (2) psycholinguistic measures are varied across the normal population (Fenson et al. 2000); (3) language disorders, which plausibly represent different breakdowns of the faculty, are very diverse by nature, and, as the relevant literature has revealed, sometimes difficult to distinguish from stages of normal language development.

All of this is appropriate for the lessons we want to draw in this article. But we think that the current revival of the biolinguistic approach has substantially expanded the realm of variation regarding language and linguistic phenomena. For example, it is now clear that it is not a handful but hundreds of genes that contribute to regulating the development and the functioning of the neural substrate of language (Benítez-Burraco 2009). Importantly, these “language genes” are polymorphic, with some variants giving rise to pathological conditions, but with others being present as well within the normal population. In fact, pathological alleles can be only regarded as such for certain populations and/or environmental conditions. Lastly, the same pathogenic allele can give rise to different language and/or cognitive disorders in different subjects. The celebrated “language gene” *FOXP2* and its interactome nicely exemplify this complex state of affairs (see Watkins 2011; Rodenas-Cuadrado et al. 2013 for reviews). Additionally, it is quite difficult to draw a precise map of the neural substrate of language, since the limits of the brain areas involved are rather changeable from one subject to another, and of course, in different clinical conditions (Fedorenko and Kanwisher 2009; Prat and Just 2011)—not to mention the additional problem that at the end of the day mapping is not explaining (Poeppel 2012). Ultimately, developmental trajectories followed by language acquisition, while displaying similar milestones, can be quite diverse, particularly at the cognitive/neurobiological levels (Bates et al. 1988; Dehaene et al. 1997). Language ontogeny in pathological populations is even more diverse, yet equally non-random (Thomas et al. 2009). It is now evident that similar cognitive profiles, in the normal population but also across pathologies, can rely on different brain architectures (Karmiloff-Smith 2010). “Modules are not born; they are made” (Bates et al. 1988, p. 284), although their basic wiring is achieved before birth, plausibly, under genetic guidance. This means that, eventually, we will have to address the diverse sources of variation (genetic, neurobiological, etc.) just mentioned in the context of a developmental perspective, allowing for different trajectories that eventually converge phenotypically.

Unfortunately, for linguists who confess a biological orientation, even those directly concerned with language development, this kind of variation “at the bottom” is ignored. They tend to idealize away from it, at their own peril. We say this because we think that the layers of variation just mentioned and the developmental dynamics that they involve lead to an important conclusion: it seems that there can be different ways of implementing a (more or less) functional faculty of language (see also Hancock and Bever 2013), and that talk of a “linguistic genotype” is fraught with difficulties.

Our main point is that the naive depictions of the biology of language that continue to dominate the literature must be improved. In our opinion, all this variation “at the bottom” can be reconciled with a certain notion of universality, but only if biolinguists are willing to engage seriously, and comprehensively, with the biology literature; i.e., only by pursuing a program that has been called biolinguistics in the strong sense of the term in Boeckx and Grohmann (2007). We wish to stress that this is not a message exclusively directed towards linguists. As we show below, a productive comparative biolinguistics also needs to take into account lessons from linguistics about the nature of language in order to develop appropriate tools for comparison.

## Which Biology Does Universal Grammar Require?

In our opinion, the root of the problem discussed in the previous section lies in the assumption that language features are directly rooted in the genome. As the literature (reviewed in Longa and Lorenzo 2008, 2012; Lorenzo and Longa 2009) reveals, a “linguistic genotype,” uniform across the species, is explicitly postulated in numerous publications. This linguistic genotype is further equated to a Universal Grammar. Ultimately, nativism is conflated with geneticism.

However, as we have already pointed out, most (if not all) “language genes” are polymorphic, with some alleles affecting language development also in the “normal” population. In addition, genes do not code for cognitive properties. A direct link between the genotype and the phenotype is not only simplistic, but biologically untenable, given the way in which genes contribute to developmental processes, and how development actually takes place. Genes are not blueprints. Developmental processes also depend on non-genetic factors (Oyama et al. 2001; Newman et al. 2006; Bateson and Mamelli 2007).

Concerning the neural substrate of language, it seems that brain areas actually perform basic kinds of computations that are recruited for different, high-level cognitive functions. As Poeppel and Embick (2005, p. 112) state, “differently structured cortical areas are specialized for performing different types of computations, and… some of these computations are necessary for language but also for other cognitive functions.” Consequently, cognitive capacities such as language are very probably cross-modular by nature. They result from the interplay of these diverse brain areas performing basic, low-level activities (Griffiths 2007). At the same time, it is only these structures that are the final output of genetically driven developmental processes. In fact, it seems that it is only their basic architecture that is genetically encoded, while their functional specificities are environmentally driven in various ways that remain to be elucidated. This is what ultimately supports the claim that modules are not born, but made, and that there is not just one way of implementing a functional language.

Our main point here is that we must seriously study how developmental dynamics, of the sort that is at the heart of EvoDevo approaches in biology (Oyama et al. 2001; West-Eberhard 2003; Callebaut and Rasskin-Gutman 2005; Carroll 2005; Müller 2007), takes place if we really want to adequately deal with this issue of variation in language, and eventually, to achieve a real biological depiction of the language faculty. In a real sense it is the complex and changing interaction between the organism and its environment that shapes the final cognitive architecture of the brain. Different factors, both internal and external, affect language development, to the extent that the different cognitive phenotypes can emerge from the same genotype (the reverse is also true: the same phenotypes can emerge from distinct genotypes). Put differently, it is clear that static depictions of language at all levels of analysis are inadequate, particularly at the biological level. In all fairness, we should point out that linguists are quite familiar with the idea that variation and change are tightly interwoven. For instance, they are well aware of the fact that language change is always preceded by a phase of variation, with different linguistic variants coexisting within the same community of speech (Weinreich et al. 1968 and much subsequent work). However, we do not find this lesson always reflected in the literature on language acquisition and developmental disorders. For us, the right position to adopt is this one: “to understand developmental outcomes, it is vital to identify full developmental trajectories, to assess how progressive change occurs from infancy onwards, and how parts of the developing system may interact with other parts differently at different times across ontogenesis” (Karmiloff-Smith 2009, p. 58).

In order to fully exploit the resources offered to biolinguistics by EvoDevo, we think that it is just as important to stress that although we have insisted on variability so far, there are, of course, many sources of universality—over and above the genes. For example, at the neurobiological level we observe that anatomical variability is quite constrained. In this way, myelinization patterns, receptor maps, cytoarchitectonic probability maps, and other structural features can be confidently established (Zilles and Amunts 2009). Similarly, functional variability seems to be constrained as well, to the extent that regions of interest can be identified (Fedorenko et al. 2010). In sum, although variation is omnipresent, the brain still exhibits a robust structure when processing language (Grodzinsky 2010). At the molecular level, we observe that the initial wiring of the linguistic brain is similarly achieved in all subjects under the guidance of a core set of genetic cues (Benítez-Burraco 2009). When it comes to language growth in the child, we find as well that developmental itineraries are also constrained although not fully predetermined, as Lenneberg (1967) already noted.

Arguably, it is the ontogeny of language disorders that more clearly reveals the real nature of the problem we want to urge linguists to wrestle with. What we recurrently observe in pathological populations is that:

1. Diffuse effects on the brain and on cognitive capacities/abilities are the norm. In fact, developmental disorders are better characterized by associations across domains than by dissociations between them (Bishop 2002).
2. Deficits in low-level, more generalized processes usually manifest as disturbances of upper, more specialized processes, which ultimately give rise to shortcomings in even higher-level, more specific cognitive capacities (Karmiloff-Smith 2009).
3. Importantly, impaired, delayed, or deviant systems are still adaptive. It is indeed worth bearing in mind that substantially preserved linguistic abilities can be achieved in spite of deeper cognitive impairments (Sirois et al. 2008; Parisse and Maillart 2009).
4. At the same time, our reading of the literature suggests to us that breakdowns and compensations, whenever they occur, do not proceed randomly. In reality, some aspects of language processing seem to be particularly vulnerable in all pathological conditions, while others seem to be preserved in all of them. For instance, inflectional morphology is problematic not just for people with specific language impairment (Marchman et al. 1999), but also for those suffering from speech-sound disorder (Mortimer and Rvachew 2010), Down’s syndrome (Eadie et al. 2002), or (a subtype of) autism (Roberts et al. 2004). Ultimately, only some pathological phenotypes have been described, while others have not been observed, a situation that we think could benefit from being modeled in terms of morphospaces or adaptative landscapes (Svensson and Calsbeek 2012). It seems, then, that although there is not just one way of implementing a linguistic brain, it is also true that there are not so many ways of implementing a functional faculty of language.

We believe that key EvoDevo concepts like canalization, developmental plasticity, robustness, evolvability, or adaptative landscapes will greatly help in clarifying, understanding, and eventually explaining the problem, and the full scope of variation in language. In all situations language development turns out to be sensitive to environmental changes, to the extent that different cognitive architectures may result from different linguistic input, as we observe in bilingual people (*developmental plasticity*). At the same time the language faculty has been shown to be remarkably resistant to (some sort of) environmental perturbations (*robustness*) to the extent that it recurrently emerges in all individuals, even in some pathological conditions (*canalization*). Moreover, some components of the language faculty seem to be very resistant to damage and/or to evolutionary change (again, *robustness*); at the same time linguistic systems seem prone to change (*evolvability*). All these properties result from the modular organization of the biological substrate of the faculty at all levels of analysis (Wagner and Altenberg 1996; Bergman and Siegal 2002; Kitano 2004). In particular, we want to suggest that developmental dynamics strongly canalizes the existing variation, to the extent that the same phenotype—i.e., a language faculty—can robustly emerge at the term of growth from diverse genotypes and brain architectures. Phenotypic uniformity, then, may be achieved *in spite of*, *and along with* neurobiological and genetic diversity.

Conceptually speaking, the state of affairs we have described in this section reminds us of the “embryonic hourglass” situation, discussed from an EvoDevo perspective in Newman (2011). As Newman observes in the context of a discussion on the evolution of animal eggs (2011, p. 467), “why can taxa within a given phylum exhibit very different egg types, pass through a common intermediate morphology (the so-called ‘phylotypic stage’), only to diverge again” (hence the metaphor of the “hourglass”)? Could the logic of the “self-organizing physical processes” that Newman relies on to answer this question also apply to situations like the one we have discussed here in the context of language? It is too early to know, but we think it is worth beginning to think about language development in this way.

We think that this EvoDevo-inspired approach to variation in language is bound to be of great interest for clinical linguistics. In particular, this distancing between the genotype and the phenotype has the potential to explain why in some people affected by a particular language disorder the sequence of the candidate genes is normal (*phenocopy*), but also why language can be preserved in individuals who are endowed with a pathogenic copy of one of these “language genes” (*null penetrance*). From a broader perspective, we are in fact tempted to argue that language disorders can be construed as conditions in which that process of canalization has failed to cope with the underlying variation (thus preventing reaching particular degrees of development). Similarly, they can be construed as decanalized states, following the model by Gibson (2009). According to this view, the pervasiveness and the high prevalence of complex genetic diseases among modern populations is a consequence of the uncovering of cryptic genetic variation resulting from the evolution of the human genome, and environmental and cultural perturbations (see Benítez-Burraco and Boeckx 2013 for additional remarks concerning this possibility and also for its implications regarding the evolution of language).

Nonetheless, for us, the crucial point is the recurrent outcome of research that suggests that breakdowns and compensations in language disorders do not occur randomly, and ultimately, that it is only certain normal, impaired, delayed, or deviant faculties of language that emerge in the course of development. We think that this situation is similar to the one that linguists often stress in their studies on language comparison: variation, though large and substantial, is not random, and appears to be confined to only certain components of grammar (Berwick and Chomsky 2011; Boeckx 2011; Boeckx and Leivada 2013a). But as we have already argued, we think that linguists err in taking the genotype to be the source of universality. And so we would like to encourage linguists to abandon this genocentric assumption and embrace the variation we find “at the bottom” by developing adequate tools to characterize it, an issue we return to in the next section.

In closing this section, we would like to ask why it should be the case that adaptability itself is limited or constrained in specific ways, to the extent that some perturbations cannot be eventually compensated by developmental dynamics. We’d like to suggest that certain cognitive processes are more vulnerable per se than others to damage or to developmental disturbances because they rely on less resilient neural networks and thus have less robust compensatory mechanisms. This would be due, we think, to their evolutionary novelty (Toro et al. 2010; Mantini et al. 2013). In fact, the most noticeable outcome of the biological study of language is that the genetic, physiological, and even cognitive mechanisms underlying language are actually robust after thousands of years of stabilizing selection (in other words, because they have a long evolutionary history), while language itself is very delicate. Probably, as suggested by Gibson (2009), the stable equilibrium observed in primates was disrupted by our evolutionary history as a species (in particular, by population bottlenecks and migratory movements; see Mellars 2006), by novel mutations, and by cultural changes. These changes brought about cognitive systems known as modern languages. But at the same time these changes may well have uncovered all that cryptic variation, decanalized the whole system, and ultimately, made language so sensitive to damage (but, we want to stress again, only to some kinds of damage). In other words, the human language faculty is easy to disturb because it is an evolutionary novelty, but at the same time it relies on robust biological mechanisms that are able to compensate many kinds of damage because they are considerably older. Plausibly, this may shed light on why our reading of the literature suggests to us that the same components of language tend to be affected in many language disorders, and why many other aspects of the linguistic phenotype are always quite preserved, and eventually, why these conditions are so prevalent among humans.

We think that this picture properly adjusts to current views of language evolution that embrace continuity and view novelty as the result of a reorganizational process rather than a product of innovative genes (West-Eberhard 2003; Müller 2010), but also with the view of language as a cognitive faculty resulting from the interface of components (cognitive, neural, genetic) otherwise not specifically linguistic (see Boeckx 2013 for review and discussion). The specificity of language would thus rely on the pervasive tendency of the components of the language faculty to interface whenever growth takes place in the presence of a suitable amount of linguistic stimuli.

## Tools for Comparative Biolinguistics

Nothing in biology makes sense except in the light of comparison. As the opening passages of Darwin’s two most famous books make clear, the business of biology is variation, variation, variation. Without variation, there can’t be any meaningful selection, any descent with modification, any origin of species. It is natural, then, to expect biolinguists to be fans of the comparative method as well. But as we have been at pains to point out above, a significant aspect of variation has been ignored by many biolinguists. To make matters worse, for much of its (recent) history, biolinguistics has been contrastive, not comparative, in the following sense: biolinguists have emphasized that language is the exclusivity of humans (and that among humans, the language faculty is uniform).

Recently, however, as De Waal and Ferrari (2010) have noted, a significant shift of perspective seems to be under way in cognitive science: the sharp contrastive character of top-down approaches is progressively being replaced by an “increased appreciation that the basic building blocks of cognition might be shared across a wide range of species” (p. 201). This bottom-up approach, seeking to establish “cognitive phylogenies” (Fitch et al. 2010), focuses on the fundamental capacities underlying larger cognitive phenomena and is more in line with the Darwinian logic of descent (Hauser et al. 2002). We think that this shift of perspective, along with the appreciation of variation and non-genetic sources of universality we have urged biolinguists to develop, provides the basis for a genuine, productive, comparative biolinguistic agenda.

To advance this new comparative research program, it is, of course, crucially important to pay attention to the tools one uses to compare. For obvious reasons, not all the tools developed by linguists are equally useful in this respect. In fact, given the modular proclivities of classical cognitive science (Piattelli-Palmarini 2001), the difficulties in exporting linguistic technology outside of the comfort zone of comparative linguists were to be expected. Not surprisingly, progress on the genetic basis of language capacities has been called “a linguist’s nightmare” (Piattelli-Palmarini and Uriagereka 2011), and although scholars have long been captivated by the parallels between birdsong and human speech and language, concrete, theoretically-informed proposals capturing the differences and the similarities across vocal learning capacities are hard to come by.

**Fig. 1:**
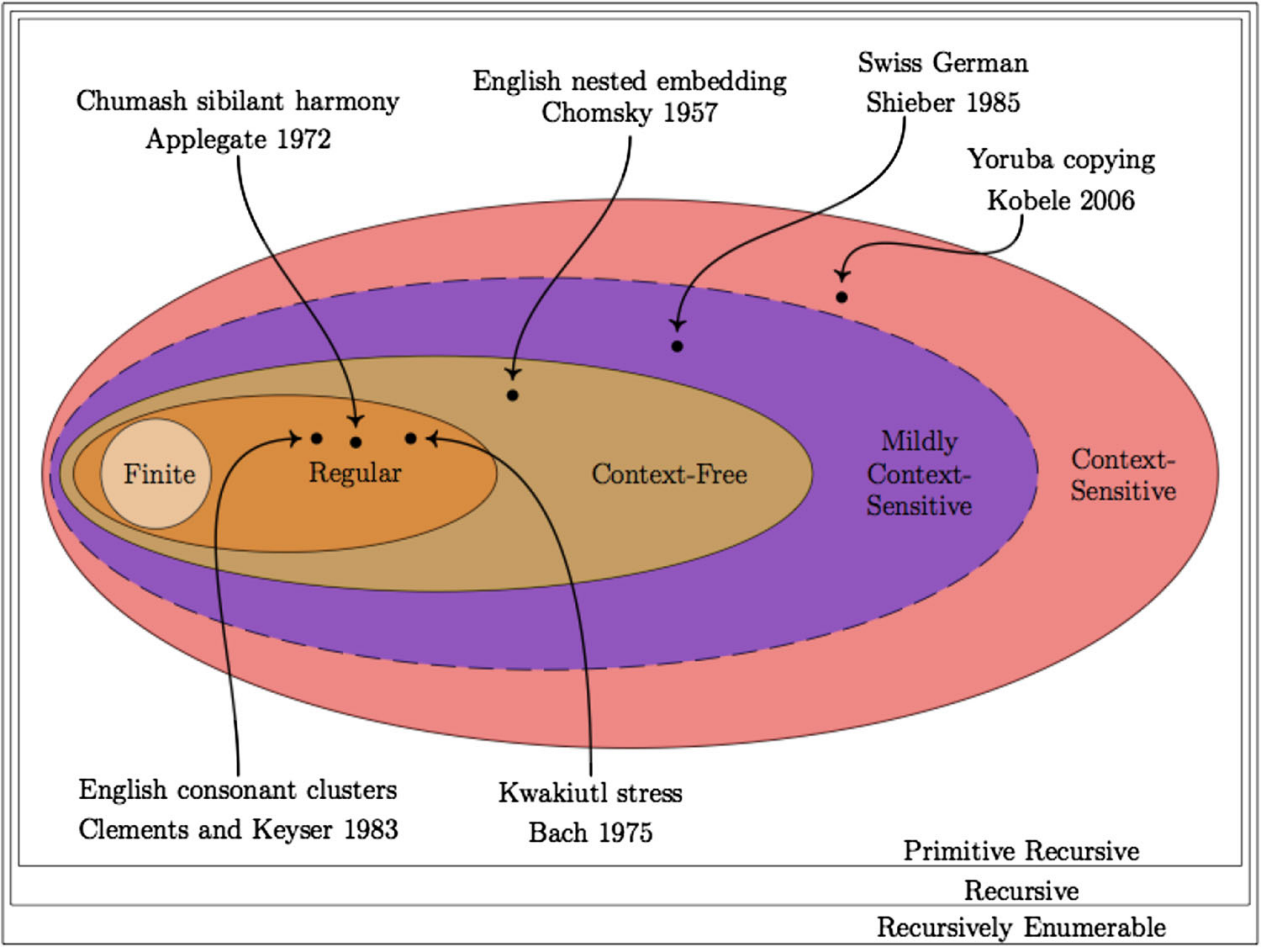
Natural language patterns in the Chomsky hierarchy; reproduced from Heinz (2014)

In the wake of Hauser et al. (2002) and the revival of biolinguistic concerns (Di Sciullo and Boeckx 2011), Fitch and Hauser (2004) were perhaps the first to face this technical challenge, and chose to resort to the hierarchy of formal languages known as the Chomsky hierarchy, developed in the 1950s, to capture the “computational constraints on syntactic processing” in non-human primates. As is well known, the Chomsky hierarchy classifies logically possible patterns into sets of nested regions. Each region corresponds to patterns describable by means of “machines” (grammars), with smaller regions captured by increasingly less powerful machinery (see Fig. 1).

As was made clear in O’Donnell et al. (2005, p. 284), the use of the Chomsky hierarchy in Fitch and Hauser (2004) was motivated by the concerns raised above:

Understanding developmental and evolutionary aspects of the language faculty requires comparing adult languages users’ abilities with those of non-verbal subjects, such as babies and non-human animals. Classically, comparative work in this area has relied on the rich theoretical frameworks developed by linguists in the generative grammar tradition. However, the great variety of generative theories and the fact that they are models of language specifically makes it difficult to know what to test in animals and children lacking the expressive abilities of normal, mature adults. We suggest that this problem can be mitigated by tapping equally rich, but more formal mathematical approaches to language.

By resorting to the Chomsky hierarchy, Fitch and Hauser hoped to avoid “theory-internal” debates corresponding to the choice of a theoretical idiom (Head-driven Phrase Structure Grammar, Government-and-Binding, Lexical-Functional Grammar, etc.) and use computational primitives that were not so language-specific as to vitiate any cross-species, or cross-domain comparison. As Heinz (2011, p. 146; emphasis in original) writes, “since *any* pattern is a language [in the sense of the Chomsky hierarchy], a distinct advantage of the Chomsky Hierarchy is [that] it allows for the comparison of patterns from different domains.”

Building on Chomsky’s (1956, 1957) foundational results concerning the limitation of finite-state machines to capture natural language generalizations, Fitch and Hauser (2004) claimed that cotton-top tamarins could not detect structures in stimuli that went beyond the computational capacity of finite-state automata. The results proved controversial in more than one way (Goudarzi 2006; Liberman 2006; Anderson 2008), but so have the results (Gentner et al. 2006; Abe and Watanabe 2011) suggesting that some songbirds outperformed cotton-top tamarins, achieving learning results beyond the finite-state boundaries (Van Heijningen et al. 2009; Berwick et al. 2011, 2012; Ten Cate and Okanoya 2012). Apart from issues of experimental design, we believe that the overarching problem lies in the adequacy of the Chomsky hierarchy in assessing cognitive profiles. Although formal language theory can certainly help in designing experiments shedding light on mental abilities (see the papers collected in Fitch and Friederici 2012), it suffers from a major problem in the context of biolinguistics. It is indeed well known that the Chomsky hierarchy is of limited use in characterizing human linguistic competence. As Berwick et al. (2012) correctly observe, the hierarchy is both “too weak and too strong,” failing as it does to cut natural language at its joints.

Heinz and Idsardi (2011, 2013) and Heinz (2014) usefully summarize important lessons that linguists have derived from applying the Chomsky hierarchy to the study of natural languages. They highlight the fact that not all natural language patterns fall exactly in the same range within the Chomsky hierarchy. In fact, as Fig. 1 reveals, patterns are quite scattered. For example, phonological patterns do not appear to require grammars that distinguish infinitely many states, unlike some syntactic patterns, which appear to require formal grammars that do. This distinction between these two domains of the language faculty highlights the fact that the human language faculty is not monolithic. It is more like a mosaic, with all the implications this has for evolutionary studies. Heinz and Idsardi also comment on the hypothesis that natural language patterns are at most mildly context sensitive (Joshi 1985) and stress that we should not conclude from this that were it true, any mildly context-sensitive pattern is automatically a possible natural language one.

There are three lessons that we would like to draw from the use of the Chomsky hierarchy in comparative biolinguistics. First, choosing to use this tool amounts to ignoring most of the research done on the nature of human languages over the past 50 years. Such research has moved away from the Chomsky hierarchy, in large part because it became clear very quickly that it does not “uniquely” characterize human language in the sense that it does not identify any (sub)region of the hierarchy as the exclusive property of natural language. As such, it does not characterize precisely enough the capacity we as humans have. Consider the fact that, as numerous linguists have observed, no natural language has rules that require counting past two. But it is a logically possible language pattern, one that the Chomsky hierarchy can capture. In fact, as Heinz (2014) notes, it is a regular pattern, falling well below the attested power range of natural language syntax. But it is a constraint that significantly shapes the human language capacity, one that we would want to ask comparative questions about. The Chomsky hierarchy does not allow us to do this. Only detailed theoretical linguistics work does.

Second, comparative biolinguistics experiments that rely on the Chomsky hierarchy tend to ignore the divide between phonology and syntax stressed by Heinz and Idsardi (2011, 2013), or rather, experimenters tend to take the notion of “syntax” too literally. The divide emphasized by Heinz and Idsardi (2011, 2013) is actually one between phonology on the one hand and syntax-semantics on the other. To the best of our knowledge, none of the evidence for syntactic patterns falling outside the regular language class comes from “pure” syntactic patterns; rather, all of them involve patterns with (structural) semantic consequences. This is an important consideration for experiments because virtually all comparative biolinguistic experiments using the Chomsky hierarchy are artificial language experiments that seek to target the learning of pure syntactic patterns (these languages don’t mean anything). But there are no such patterns in natural languages. Not surprisingly, when semantic cues are added to the experiment, as in Fedor et al. (2012), these were found to boost the learning of more complex formal grammars in humans. By ignoring semantics, artificial language experiments may well be removing the component that gives natural language syntax its distinctive computational signature.

The third, and perhaps most important, lesson that we’d like to draw is that it is not at all clear what the expectations of the relevant experiments are, due to the inherent limitations of the Chomsky hierarchy in capturing the true nature (i.e., the constraints) of natural languages. This is not to say, of course, that such experiments are pointless. They can tell us many things, but it is not clear that they allow us to draw solid conclusions concerning the system linguists call natural languages. To make ourselves clear, suppose we found out that a non-human species were capable of mastering a mildly-context-sensitive language in the context of an artificial language experiment. What would we be able to conclude from this? Our answer is, not much. In fact, we would *only* be able to conclude that they were able to learn this pattern. But it would not immediately tell us the underlying algorithm used. As reviewed in Ojima and Okanoya (2013), all the artificial grammar experiments to date suffer from this problem, as there is more than one way to acquire a particular pattern. (Remember that one of the strengths of the Chomsky hierarchy is that it allows for the comparison of patterns from different domains. But this is also its weakness—it is too nonspecific to exclude alternative cognitive ways of capturing a given pattern.)

As should be clear by now, even if a non-human species were capable of mastering a mildly-context-sensitive language in the context of an artificial language experiment, we would not be able to conclude anything regarding the human language faculty. Because there is no (sub)region of the Chomsky hierarchy that is exclusively occupied by natural languages, we would not be able to conclude that the success of non-human primates shows that they have a component of the human language faculty. It is for this reason that we are skeptical about the use of the Chomsky hierarchy to identify language regions in the brain (Fitch and Friederici 2012; Moro and Chesi in 2014) or to draw linguistic inferences from artifacts in the fossil record (Camps and Uriagereka 2006; Balari and Lorenzo 2013; Longa 2013).

As Boeckx (2013) observed, finding a substitute, or complement to the Chomsky hierarchy to construct cognitive phylogenies will be a serious challenge for the years to come. In the remainder of this section, we would like to sketch a possible research avenue that seems to us to have the right properties, and that connects to some EvoDevo concerns discussed above, as it draws on aspects of our biology that are conserved across species, and at the same time that are known to vary across human populations (language disorders).

To begin with, we’d like to step back and consider what we believe was the main factor behind the renewed interest in the comparative method in linguistics at the end of the 1970s. The “new comparative syntax,” as it has been called, grew out of proposals articulated in Chomsky (1981). These proposals, as Chomsky acknowledged on numerous occasions (e.g., Chomsky 2009), grew out of reflections inspired by the work of Jacob and Monod (1961) on gene regulation. In the fullness of time the Jacob-Monod model developed into EvoDevo genomics (Carroll 2005). The most iconic finding of this field is the hox gene set, which confirmed Monod’s prediction that “what is true for *E. coli* is also true for the elephant.” As is now well established, the set of genes regulating development across a wide range of species is shared, which greatly enhances the possibilities of comparison. Against the background of this deep conservation, species differences (variations) can be thought of as little tweaks and nudges, like the 30 variations of the aria that Bach offered us in what is now known as the Goldberg variations. Arthur (2004), for instance, suggests that all variations reduce to instances of heterochrony (different timing of gene expression), heterotopy (different location of gene expression), heterometry (more of the gene product being made), and heterotypy (change in the nature of the gene product; e.g., switch on different target genes).

This model to understand variation was borrowed into linguistics, where it came to be known as the Principles-and-Parameters approach, with the principles providing the underlying uniformity and the parameters the sources of the surface variations (Chomsky 1981; Baker 2001). The analogy worked well for two decades, allowing for considerable empirical progress, but in recent years, the foundational assumptions of the Principles-and-Parameters model have been questioned (Newmeyer 2004, 2005; Boeckx 2011, 2014; Boeckx and Leivada 2013a). It appears that in order to capture the variation that comparative linguists focus on, something else is needed, perhaps something along the lines of Boeckx and Leivada (2013b). However, the logic of Principles and Parameters may be just what is needed in the context of comparative biolinguistics. Such a model need not require genes to provide the relevant parameters (even for biology, genes may be followers, not just leaders; Newman and Bhat 2009; Schwander and Leimar 2011), but its logic demands that one find an aspect of deep conservation on which variants could be grafted.

Buzsáki et al. (2013) may provide just what is needed in this context. They observe that despite the several-thousandfold increase of brain volume during the course of mammalian evolution, the hierarchy of brain oscillations (brain rhythms) remains remarkably preserved. This conserved aspect of our biology is directly relevant for comparative biolinguistics: it offers the possibility of conceiving of cross-species differences, or, as Buzsáki et al. (2013) discuss, of cognitive diseases, as slight variations (disruptions) within the preserved network constellation that would constitute a universal brain syntax (Buzsáki 2010)—dysrhythmias and oscillopathies, as Buzsáki et al. (2013) call them. Put differently, the preservation of brain rhythms in mammals would be the cognitive scientist’s hox genes.

Obviously, to put this hypothesis to the test in the context of language, it is necessary for linguists to translate their findings concerning the properties of the human language faculty in terms of brain rhythms, to offer a mind/brain model on which to formulate parameters giving rise to distinct cognitive profiles. This translation step may, in fact, be independently necessary to bridge the gap between mind and brain. David Poeppel has written eloquently and accessibly about the challenges neurolinguistics faces (Poeppel 2005, 2011, 2012; Poeppel and Embick 2005). The heart of the matter, according to Poeppel, is the “granularity mismatch” (or “mapping”) problem: the objects of study in theoretical linguistics and in neuroscience don’t match. As a result, mapping one onto the other has proven impossible. Accordingly, Marr’s (1982) vision of cognitive neuroscience based on linking levels of analyses, to which biolinguistics should aspire, remains distant.

Both theoretical linguistics and the neurosciences are to blame for this sorry state of affairs. For all the “bio” talk in linguistic circles, linguists have so far failed to distill what is known from linguistic theory into a set of computational primitives, and to try to link these with models and specific principles of neural computation. As has been said,

we need linguistic models that are explicit about the computational primitives (structures and operations) they require, and that attempt to define linguistic problems at a fine enough grain that one can discuss algorithmic and implementational approaches to their solution. We need a list of computations that linguistic theorists deem indispensable to solve their particular problem (e.g., in phonology, syntax, or semantics). (Fitch 2009, p. 298)

Put another way,

[l]inguists and psycholinguists owe a decomposition (or fractionation) of the particular linguistic domain in question (e.g., syntax) into formal operations that are, ideally, elemental and generic. Generic formal operations at this level of abstraction can form the basis for more complex linguistic representation and computation. (Poeppel 2005, p. 11)

A rhythm-based model may have just the right kind of characteristic envisaged by Poeppel. In fact, direct evidence of the fruitfulness of this approach in the language domain comes from Poeppel’s own work, beginning with Poeppel (2003) and culminating with Giraud and Poeppel (2012).

What Poeppel and colleagues have shown is that by focusing on the endogenous rhythms generated by the cortex, it is possible to understand (as opposed to merely localizing) the cerebral specialization for speech perception and production, and to shed light on the nature of phrasal phonology. The main thesis is that neuronal oscillations contribute to cognition in several ways: for example, by segregating information and organizing spike timing. Specifically, a series of oscillations (in the delta, theta, and gamma ranges) appear to be able to track the dynamics of speech. In doing so, they “chunk” or “package” incoming information into units of the appropriate temporal granularity. This packaging corresponds to units of phrasal phonology (linking the algorithmic and computational levels). Taking linguistic processes as involving multiple subprocesses with different characteristics, as opposed to being monolithic, each one associated with different frequencies of neural oscillations, may offer us the right component parts to identify what goes wrong in disorders, or how different orchestrations may give rise to cross-species cognitive differences, and endophenotypes.

The translation work needed will be slow, of course, but the fact that we already have working candidate models of the right format for some aspects of language like phonology (Giraud and Poeppel 2012), for which we have good animal models (vocal learners) as well as a growing amount of genetic information (the FOXP2 interactome), suggests that the first fruits of a comparative biolinguistics may not be too distant.

## Conclusion

It is true that one finds less genetic variation in our species than in our cousins (a conclusion reinforced by the recent sequencing of the genomes of a large number of great apes from across Africa and Southeast Asia; Prado-Martínez et al. 2013), but we should not idealize away from the variation that nonetheless exists. That variation is real, and contains important lessons concerning the biological foundations of the human language faculty. In fact, we have argued here that this variation is only problematic for the naive—and unfortunately, still dominant—conceptions of the biological underpinnings of language in the language sciences. While there are many more sources of variation than those linguists tend to study, there are also many more sources of universality that canalize this variation and allow for languages to reliably develop in the individual than linguists tend to assume. We have furthermore suggested that lessons from comparative and theoretical linguistics may profitably be extended to deeper layers of variation, and could offer a starting point for a new branch of comparative linguistics, one we have called comparative biolinguistics. This new subfield may help correct the exuberant (genocentric) nativism that appears to be so problematic (Boeckx and Leivada 2013a), particularly for achieving a fruitful assimilation of linguistics to biology, allowing for a real, biologically grounded biolinguistics to emerge.
